# 2219. Guiding stewardship interventions through tracking of bone cultures in diabetic lower extremity infections

**DOI:** 10.1093/ofid/ofad500.1841

**Published:** 2023-11-27

**Authors:** Pawlose Ketema, Ayne Adenew, Angela Y McKnight, Janette Thompson, Angelike P Liappis

**Affiliations:** Veterans Affairs (VA) National Capitol Care Network (VISN5) Antimicrobial Stewardship Clinical Collaborative of VA Medical Centers in Washington DC/Martinsburg WV/Baltimore MD/Clarksburg WV/Beckley WV/Huntington WV, Washington, District of Columbia; Washington DC Veterans Affairs, Washington, District of Columbia; DC VA Medical Center, Washington, District of Columbia; Washington DC Veterans Affairs, Washington, District of Columbia; Washington DC Veterans Affairs Medical Center , Washington, DC

## Abstract

**Background:**

Lower extremity infections (LEIs) of diabetic patients poses a challenge to addressing over-utilization of broad spectrum antibiotics (BSA). To guide the development of educational and behavioral initiatives, we employed ASP quality improvement surveillance practices to explore podiatric-surgery collected bone cultures (BC), pre-procedural tissue cultures (PTC) and results of resection margin pathology.

**Methods:**

Washington DC VAMC is an urban medical center with 220 acute and LTC beds with acute and outpatient surgical Podiatry Service and supported by an active ASP (PharmD/MD/NP). Between 2020-2022 positive BCs collected in podiatric LEI procedures for presumed osteomyelitis were reviewed by ASP with CDSS (TheraDoc, Inc) and EMR (VISTA_CPRS). A positive post-resection margin (Margin+), empiric antibiotic selection and microbiology including regimen altering organisms such as MRSA and P. aeruginosa (PSAR) were collected.

**Results:**

Diabetic individuals had 94 positive BCs in 296 procedures among nearly 2,000 total cultures. Subjects were predominately male (97%), mean age 68 ± 8.7 (45-93y); and nearly a third had > 1 BC. Intraoperative BCs made up 79% of episodes and half were Margin+. Subjects had high rates of PVD (45%), insulin-dependence (49%) and HbA1c > 10 mg/dL (21%). Empiric BSA selection consisted mainly of MRSA and PSAR coverage with no deaths < 30d from BC. Among BC with PTC, 61% had partial or complete correlation in microbiology; MRSA and PSAR incidence were the same if PTC:BC findings were concordant or not. PTC:BC microbiologic non-concordance vs concordance for Margin+ was not significantly different between the groups (45.8% vs 59.4%, P=NS) and among PTC:BC concordant samples, PSAR rates trended higher (12.9% vs 4.8%, P=NS) with no difference in MRSA incidence.

**Conclusion:**

Providers have limited information in predicting LEI results in advance of path and micro. Empiric BSAs remains a challenge for ASPs. Less than half of patients had a BC finding that would have been predicted by the PTC and for those with a concordant prior to procedure sample, the finding of organisms that directed the empiric selection were not reflective of final cultures. There are opportunities for ASP interventions that address empiric selection in diabetic LEIs.

**Disclosures:**

**All Authors**: No reported disclosures

